# Expanding the donor pool: Outcomes of liver transplantation using grafts with ≥50% macrosteatosis

**DOI:** 10.1016/j.liver.2025.100311

**Published:** 2025-12-08

**Authors:** Kasra Shirini, Shani S. Kamberi, Srinivasan Muthukrishnan, Omar Alattar, Ruchin Patel, Francheska Nieves, Massimo Arcerito, Josue Alvarez-Casas, Saad Malik, Kirti Shetty, Daniel G. Maluf, Chandra Bhati, Raphael P.H. Meier

**Affiliations:** Division of Transplant Surgery, Department of Surgery, University of Maryland School of Medicine, Baltimore, MD, USA

**Keywords:** Liver macrosteatosis, Liver transplantation, Liver graft, Transplantation

## Abstract

Liver grafts with over 50% macrosteatosis are often deemed marginal, but organ shortages necessitate exploring their use. We conducted five transplants with deceased donor grafts containing 50%–90% macrosteatosis, ensuring donor age <55 years, CIT ≤6 hours, and recipient laboratory MELD score ≤30. Two patients required reoperations due to thrombotic complications (acute HAT and PVT), and hospital stay ranged from 6 to 26 days. All patients survived beyond one year with functioning grafts. These findings suggest the feasibility of using high-macrosteatosis grafts in intermediate- to low-laboratory-MELD recipients, albeit with potential thrombotic risks.

## Introduction

Metabolic dysfunction–associated fatty liver disease (MAFLD) impacts up to 50 % of the global population, contributing to a growing pool of steatotic liver grafts [1]. Donor livers with ≥50 % macrosteatosis are often considered marginal grafts, with ≥55 % discarded despite evidence showing acceptable long-term outcomes when appropriately selected and managed [2]. While moderate macrosteatosis (30–60 %) is associated with higher early mortality due to complications such as postreperfusion syndrome, cardiac arrest, and acute kidney injury, it does not significantly affect long-term survival, with 5- and 10-year outcomes comparable to non-steatotic grafts [3]. Steatotic grafts remain a valuable resource when managed with optimized protocols. We hypothesized that strict recipient-donor selection criteria could enable the successful transplantation of high macrosteatosis (≥50 %) livers into low-risk recipients. We present five cases demonstrating this approach and achieving acceptable post-transplantation outcomes. Eligible liver grafts were defined by ≥50 % macrosteatosis in cases of young brain death donors (ideally age<55), peak AST/ALT ≤500/350, projected cold ischemia time ≤6 h, and recipients with laboratory MELD scores ≤30. Graft steatosis was quantified on hematoxylin and eosin frozen sections by an independent liver pathologist and confirmed by our own liver pathologist. Median with range is presented. This study was conducted in accordance with the Declaration of Helsinki and approved by the University of Maryland, Baltimore Institutional Review Board (protocol HP-00,113,760). All recipients provided informed consent.

## Description of cases

### Recipients

**Fig. 1.**
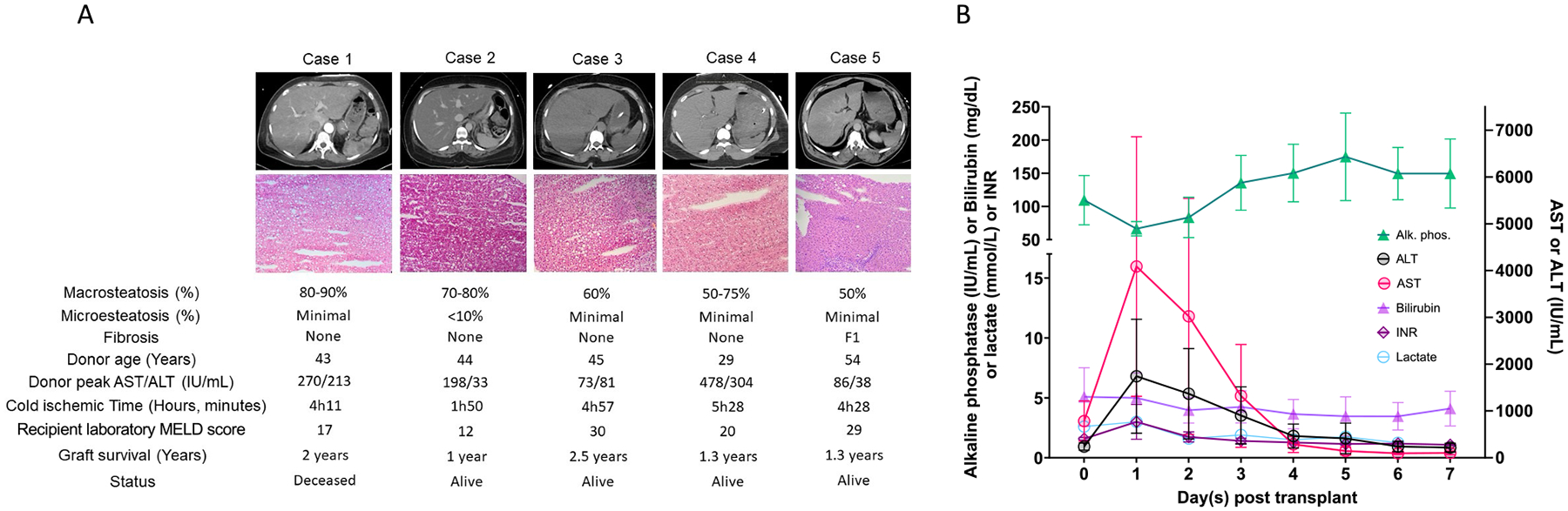
**A**. Summary of five transplant cases, including abdominal CT-scan, microscopic liver tissue, donor age, cold ischemia time, macrosteatosis, fibrosis, graft survival, and recipient status. **B**. Recipient postoperative labs from day 0 to 7.

Five patients underwent transplantation following our protocol (Fig. 1.A). The median recipient age was 61 (40 – 72) years, with a BMI of 27.1 (19.9 – 30.8) kg/m^2^ (Table 1). Laboratory MELD scores ranged from 12 to 30, with a median of 20. Two cases had laboratory MELD scores of 29 and 30, stable hemodynamics, preserved renal function, and no signs of infection. The primary indications for transplantation included cirrhosis due to chronic hepatitis C associated with hepatocellular carcinoma (HCC) in just one case, metabolic dysfunction-associated steatohepatitis (MASH) (2 cases), and alcohol-associated liver disease (ALD) (2 cases). No macrovascular tumor thrombus was detected on preoperative imaging or biopsy. None of the other recipients had a history of HCC. All patients underwent comprehensive preoperative assessments, including abdominal CT imaging and liver biopsies, to confirm the underlying pathology and evaluate transplant candidacy.

### Donors

The donors had an average age of 43 ± 9 years and a mean BMI of 35.1 ± 3.1 kg/m^2^. macrosteatosis levels averaged 66.5 ± 13.6 % (range: 50–90 %), with minimal microsteatosis (<10 %) in all cases. Fibrosis was limited to F1 in one donor liver, with no significant fibrosis in the others. Causes of donor death included anoxia (3 cases), cerebrovascular stroke (1 case), and meningoencephalitis (1 case). Donor liver enzymes showed peak AST/ALT values of 198 (73 – 478) IU/L and 81 (33 – 304) IU/L, respectively, with terminal values of 53 (17 – 276) IU/L and 33 (28 – 110) IU/L. The median cold ischemia time (CIT) was 4h28, ranging from 1h50 to 5h28.

### Operation details

During transplantation, vascular anastomoses were completed in a median of 39 (36 – 55) minutes. Estimated blood loss was 2.5 (2 – 12) L, with patients receiving a median of 6 RBC units. One case of severe reperfusion injury was observed in case 1. This patient was relisted and placed on MARS but ultimately did not need a retransplant and fully recovered.

### Postoperative outcomes

Postoperative liver function tests showed peak AST and ALT levels of 2425 (571 – 15,000) IU/L and 400 (297 – 6559) IU/L, respectively, normalized within four days (Fig. 1.B). Two patients developed prothrombotic complications: one case of portal vein thrombosis (PVT), and one case of hepatic artery thrombosis both managed surgically. Additionally, two patients required reoperations due to biliary leakage.

### Follow-up and survival

The median hospital stay was 11 days, ranging from 6 to 26 days. Median follow-up was 476 (396 – 920) days. The 2-year graft survival rate was 100 %, with four patients alive and maintaining well-functioning grafts at the end of the follow-up. One patient passed away after two years due to HCC recurrence.

## Discussion

We demonstrate the feasibility of transplanting macrosteatosis liver allografts (≥50 %) with acceptable long-term outcomes, conditional on a careful donor-recipient selection. Our protocol prioritized low-risk recipients with low laboratory MELD scores, short CIT, and young donors with low AST/ALT values, aligning with prior studies showing that meticulous graft selection reduces early dysfunction risks [2].

Low laboratory MELD scores, young donor age, and short CIT are crucial for successful steatotic graft transplantation. In our series, two patients with laboratory MELD scores of 29 and 30 were included. The absence of aggravating risk factors such as severe renal dysfunction, severe portal hypertension, or systemic infection allowed us to proceed with these cases. Kaltenbach et al. evidenced that macrosteatosis over 40 % raises short-term risks but not long-term outcomes within high-risk subgroups [4]. A 2025 multicenter study found that steatotic grafts in liver transplantation for HCC were associated with worse disease-free survival and overall survival compared to non-steatotic grafts, particularly with longer cold ischemia times (≥6 h), higher donor BMI (≥40 kg/m^2^), and recipients with non-alcoholic fatty liver disease, included in the HAML score [5]. Of note, none of our recipients had any of these risk factors.

Our recipients showed high post-transplant AST/ALT peak levels, indicating significant Ischemia-reperfusion injury (IRI). Considering this, we prioritized donors with moderately elevated AST/ALT values to minimize the double hit. Notably, sequential biopsy studies have demonstrated that moderate macrosteatosis (30–60 %) often resolves rapidly post-transplant, with complete regression observed within one week and sustained at six months. This supports our observation that steatosis may not persist long-term if recipients overcome the early perioperative risks [6].

Steatotic liver grafts are particularly vulnerable to IRI due to increased mitochondrial oxidative stress, impaired ATP-restoration, and heightened inflammatory responses [7]. Despite these challenges, satisfactory outcomes were achieved without machine perfusion. These findings align with broader reviews of marginal grafts, which emphasize that steatotic livers—along with elderly and DCD grafts [8–10]—can expand the donor pool when paired with optimized donor–recipient matching and preservation strategies [11]. *Ex vivo* hypothermic and normothermic machine perfusion (NMP) is increasingly used in the U.S. to reduce IRI, improve graft survival, and mitigate biliary complications [12,13]. We did not have access to machine perfusion at that time; however, it is likely that the liver grafts we describe here would have benefited from being pumped and further assessed. So-called ‘defatting protocols’ offer further potential for optimizing extreme steatotic grafts [14]. These protocols leverage pharmacological agents such as forskolin (NKH477), L-carnitine, and insulin-like growth factor-1 (IGF-1) to reduce triglyceride content and restore mitochondrial function [15]. Recent studies have shown that ‘defatting protocols’ during NMP can reduce fat content by up to 40 % in severely steatotic livers, enhancing graft viability and expanding the donor pool [15]. While promising, clinical evidence remains limited; further studies are needed to validate their safety and efficacy.

Our case series underscores the feasibility of utilizing severely macrosteatotic grafts, encouraging their broader acceptance in carefully selected recipients. Stringent postoperative monitoring is crucial for the early detection of early allograft dysfunction (EAD) and primary non-function. Various parameters and scoring systems can be used to closely monitor the recipient of a severely steatotic liver graft [16,17].

Vascular complications were notable in our study, with one patient developing hepatic artery thrombosis and another experiencing PVT, both managed successfully. One may hypothesize that the cytokine storm following steatotic graft implantation might favor prothrombotic conditions, possibly compounded by ischemia–reperfusion injury and endothelial dysfunction. These complications contributed to extended hospital stays and which highlights the need for careful perioperative management. While no consensus exists on prophylactic anti-coagulation, its selective use in high-risk cases, such as those with prior PVT or complex anastomoses, hypercoagulable states, warrants further consideration.

Our report is limited by its small sample size, variability in sampling and histologic steatosis evaluation, lack of a control group, and medium-term follow-up. These limitations underscore that our findings should be regarded as descriptive and primarily hypothesis-generating. Future multicenter studies are needed to validate findings, evaluate the recurrence of hepatic steatosis or progression to NASH, and assess the utilization of steatotic grafts in the setting of advanced preservation techniques. ‘Defatting protocols’ combined with machine perfusion could optimize the utilization rate of steatotic grafts.

In conclusion, transplanting macrosteatosis liver grafts in selected recipients is feasible. Despite important limitations such as small sample size, lack of a control group, and variability in histologic evaluation, our findings suggest that, with strict selection criteria, these grafts can expand the donor pool while maintaining excellent liver transplantation outcomes.

## Figures and Tables

**Table 1:** Comparison of recipient and donor characteristics across five liver transplant cases.

	Characteristic	Recipients					
		Case 1	Case 2	Case 3	Case 4	Case 5	Median (Min-Max)
Recipient	**Gender**	Male	Male	Male	Male	Male	N/A
	**Age at transplant (years)**	67	72	58	61	40	61 (40 – 72)
	**BMI (Kg/m** ^**2**^ **)**	30.8	27.7	27.1	30.3	19.9	27.1 (19.9 – 30.8)
	**MELD score**	17	12	30	20	29	20 (12 – 30)
	**Cirrhosis etiology**	HCV/HCC	MASH	EtOH	MASH	EtOH	N/A
	**Hepatocellular carcinoma**	Yes	No	No	No	No	N/A
	**Portal vein thrombosis at transplant**	Yes	Yes	No	No	No	N/A
	**Anastomosis time (minutes)**	45	55	38	39	36	39 (36 – 55)
	**Operating time (hours, minutes)**	4 h 56m	2 h 45m	5 h 35m	6 h 7m	5 h 4m	5h4m (2h45m – 6h7m)
	**RBC units received**	5	6	7	6	9	6 (5 – 9)
	**Estimated blood loss (liter)**	2.0	8.0	2.5	12.0	2.5	2.5 (2.0 – 12.0)
	**MARS**	Yes	No	No	No	No	N/A
	**Peak AST (IU/L)**	15,000	2425	571	729	3205	2425 (571 – 15,000)
	**Peak ALT (IU/L)**	6559	385	297	400	1216	400 (297 – 6559)
	***Re*-operation**	Yes	Yes	No	No	No	N/A
	**Post-operative complications**	PVT	HAT/ Biliary leak	Open wound foot	AKI, fever	Fever, tachycardia/ fall, fever	N/A
	**Post-op LOS (days)**	26	6	11	15	11	11 (6 – 26)
	**Last follow-up status**	Dead	Alive	Alive	Alive	Alive	N/A
	**Follow-up (days)**	762	396	920	476	471	476 (396 – 920)
Donor	**Donor type**	DBD	DBD	DBD	DBD	DBD	N/A
	**Age (years)**	43	44	45	29	54	44 (29 – 54)
	**BMI (Kg/m** ^**2**^ **)**	31.7	35.3	32.8	39.7	36.1	35.3(31.7 – 39.7)
	**Cause of death**	Anoxia	Meningoencephalitis	Anoxia	Anoxia	Cerebrovascular accident	N/A
	**Arterial anatomy**	Early take-off of RHA from celiac	Standard	Standard	Standard	Standard	N/A
	**Cold ischemia time (hour, minutes)**	4h11m	1 h 50m	4 h 57m	5 h 28m	4 h 28m	4h28m (1h50m – 5h28m)
	**Peak AST (IU/L)**	270	198	73	478	86	198 (73 – 478)
	**Peak ALT (IU/L)**	213	33	81	304	38	81 (33 – 304)
	**Terminal AST (IU/L)**	27	198	17	276	53	53 (17 – 276)
	**Terminal ALT (IU/L)**	48	33	28	110	33	33 (28 – 110)
	**Terminal bilirubin (mg/dL)**	0.5	0.4	0.7	0.3	1.2	0.5 (0.3 – 1.2)
	**Terminal ALP (IU/L)**	75	137	62	95	101	95 (62 – 137)
	**Terminal INR**	1.2	1.1	1.6	1.5	1.1	1.2 (1.1 – 1.6)
	**Macrosteatosis (%)**	80–90 %	70–80 %	60 %	50–75 %	50 %	N/A
	**Microesteatosis (%)**	Minimal	<10 %	Minimal	Minimal	Minimal	N/A
	**Fibrosis**	None	None	None	None	F1	N/A

Abbreviations: AKI: Acute Kidney Injurie, ALP: Alkaline phosphatase, ALT: Alanine Aminotransferase, AST: Aspartate Aminotransferase, BMI: Body Mass Index, EtOH: Ethanol, HAT: Hepatic Artery Thrombosis, HCV: Hepatitis C Virus, HCC: Hepatocellular carcinoma, INR: International normalized Ratio, LOS: Length of Stay, MARS: Molecular Adsorbent Recirculating System, Max: Maximum, MELD: Model of End-Stage Liver Disease, Min: Minimum N/A: Not Applicable, NASH: Non-alcoholic steatohepatitis, PVT: Portal Vein Thrombosis, RBC: Red Blood Cells, RHA: Right Hepatic Artery.

## Data Availability

The data that support the findings of this study are available on request from the corresponding author.

## Abbreviations:

AKI: acute kidney injury
ALD: alcohol-associated liver disease
ALP: alkaline phosphatase
ALT: alanine aminotransferase
AST: aspartate aminotransferase
BMI: body mass index
CIT: cold ischemia time
CT: computed tomography
DBD: donation after brain death
EAD: early allograft dysfunction
EBL: estimated blood loss
EtOH: ethanol/alcohol
HAT: hepatic artery thrombosis
HCC: hepatocellular carcinoma
HCV: hepatitis C virus
IGF-1: insulin-like growth factor-1
INR: international normalized ratio
IRI: ischemia-reperfusion injury
IU/L: international units per liter
LOS: length of stay
MAFLD: metabolic dysfunction-associated fatty liver disease
MARS: Molecular Adsorbent Recirculating System
MASH: metabolic dysfunction-associated steatohepatitis
MELD: Model for End-Stage Liver Disease
NASH: non-alcoholic steatohepatitis
NMP: normothermic machine perfusion
PVT: portal vein thrombosis
RBC: red blood cells
RHA: right hepatic artery
